# Cardiac MR modelling of systolic and diastolic blood pressure

**DOI:** 10.1136/openhrt-2023-002484

**Published:** 2023-12-18

**Authors:** Hosamadin Assadi, Gareth Matthews, Xiaodan Zhao, Rui Li, Samer Alabed, Ciaran Grafton-Clarke, Zia Mehmood, Bahman Kasmai, Vaishali Limbachia, Rebecca Gosling, Gurung-Koney Yashoda, Ian Halliday, Peter Swoboda, David Paul Ripley, Liang Zhong, Vassilios S Vassiliou, Andrew J Swift, Rob J van der Geest, Pankaj Garg

**Affiliations:** 1 Department of Cardiovascular and Metabolic Health, University of East Anglia, Norwich, UK; 2 Norfolk and Norwich University Hospitals NHS Foundation Trust, Norwich, UK; 3 National Heart Research Institute, National Heart Centre, Singapore; 4 Department of Infection, Immunity & Cardiovascular Disease, University of Sheffield, Sheffield, UK; 5 LICAMM, University of Leeds, Leeds, UK; 6 Department of Cardiology, Northumbria Specialist Emergency Care Hospital, Cramlington, UK; 7 Cardiovascular Science Academic Program, Duke-NUS Medical School, Singapore; 8 Department of Radiology, Leiden University Medical Center, Leiden, The Netherlands

**Keywords:** Magnetic Resonance Imaging, Hypertension, Aortic Diseases, Cardiac Imaging Techniques

## Abstract

**Aims:**

Blood pressure (BP) is a crucial factor in cardiovascular health and can affect cardiac imaging assessments. However, standard outpatient cardiovascular MR (CMR) imaging procedures do not typically include BP measurements prior to image acquisition. This study proposes that brachial systolic BP (SBP) and diastolic BP (DBP) can be modelled using patient characteristics and CMR data.

**Methods:**

In this multicentre study, 57 patients from the PREFER-CMR registry and 163 patients from other registries were used as the derivation cohort. All subjects had their brachial SBP and DBP measured using a sphygmomanometer. Multivariate linear regression analysis was applied to predict brachial BP. The model was subsequently validated in a cohort of 169 healthy individuals.

**Results:**

Age and left ventricular ejection fraction were associated with SBP. Aortic forward flow, body surface area and left ventricular mass index were associated with DBP. When applied to the validation cohort, the correlation coefficient between CMR-derived SBP and brachial SBP was (r=0.16, 95% CI 0.011 to 0.305, p=0.03), and CMR-derived DBP and brachial DBP was (r=0.27, 95% CI 0.122 to 0.403, p=0.0004). The area under the curve (AUC) for CMR-derived SBP to predict SBP>120 mmHg was 0.59, p=0.038. Moreover, CMR-derived DBP to predict DBP>80 mmHg had an AUC of 0.64, p=0.002.

**Conclusion:**

CMR-derived SBP and DBP models can estimate brachial SBP and DBP. Such models may allow efficient prospective collection, as well as retrospective estimation of BP, which should be incorporated into assessments due to its critical effect on load-dependent parameters.

**WHAT IS ALREADY KNOWN ON THIS TOPIC**

- Prior research has established the significance of blood pressure (BP) in cardiovascular health and its potential influence on cardiac imaging assessments. It is recognised that accurate BP measurements are essential for a comprehensive understanding of cardiovascular function. However, conventional outpatient cardiovascular MR (CMR) imaging procedures typically do not involve BP measurements before image acquisition. This gap in the field highlights the need for a method to estimate BP using CMR data and patient characteristics, which is the focus of this study.

**WHAT THIS STUDY ADDS**

- This study adds a novel approach to the field by proposing a model to estimate brachial systolic and diastolic BP using patient characteristics and CMR imaging data. By demonstrating associations between specific CMR parameters and BP, the study provides a potential means to collect BP data prospectively during CMR imaging efficiently and retrospectively estimate BP values. This approach fills a crucial gap in standard CMR procedures, allowing for a more comprehensive assessment of cardiovascular health by incorporating BP, which is known to impact load-dependent parameters significantly.

**HOW THIS STUDY MIGHT AFFECT RESEARCH, PRACTICE OR POLICY**

- This study’s novel approach to estimating could revolutionise cardiovascular research by streamlining data collection, enhancing the precision of cardiac imaging assessments and facilitating retrospective BP estimation. It may guide treatment decisions and risk assessment in clinical practice, potentially influencing healthcare policies to incorporate BP measurements into CMR protocols. Ultimately, it can potentially elevate the standard of cardiovascular care by integrating BP measurements into CMR protocols.

## Introduction

Blood pressure (BP) plays a vital role in haemodynamic assessment. Hypertension is the most common preventable risk factor for cardiovascular disease and is a major contributor to all-cause mortality, given its pathogenic role in stroke and chronic kidney disease. Worldwide, an estimated 31.1% of adults have hypertension, with an increased prevalence in low-income and middle-income countries.^1^ Physiologically, BP is dependent on numerous factors, including heart rate, stroke volume, systemic vascular resistance, blood volume, arterial compliance and neuroendocrine axes.^2^ In most cases, hypertension is straightforward to address with lifestyle and pharmacological strategies. Hypotension can also cause morbidity and mortality, particularly in older adults and those with heart failure.^3 4^

From a cardiac imaging perspective, BP influences the evaluation of ventricular size, chamber function and severity of valve pathology during functional assessment.^5–7^ Hypertension is commonly present in heart failure,^8^ especially in the preserved ejection fraction phenotype, left-sided valvular disease,^9^ ischaemic heart disease and atrial fibrillation. Numerous imaging guidelines incorporate the assessment of loading conditions to accurately grade specific lesions, with changes in loading conditions recognised as potential causes for misclassification of disease severity.^10 11^ BP is dynamic and impacted by body positioning and activity. Standard outpatient guidelines recommend BP assessment after a patient is seated and relaxed with feet on the floor for more than 5 min. Furthermore, to minimise random error, 2–3 BP measurements should be obtained on 2–3 different occasions.^12^

In standard outpatient cardiovascular MR (CMR) imaging procedures, BP measurement is usually not taken prior to image acquisition. As a result, during routine CMR examinations, it is challenging to make a comprehensive afterload-dependent assessment of load-dependent imaging. Moreover, CMR is emerging as a prognostically relevant test for aortic and mitral valve diseases, particularly mitral regurgitation and aortic regurgitation—both dynamic valvular incompetencies with dependence on BP. The time efficiency of CMR protocols is improving; however, brachial BP recording would increase radiographers’ workload and workflow durations. Additionally, for retrospective analysis, there is presently no method to infer BP and, therefore, loading conditions in the absence of contemporaneous measurement.

We hypothesise that both systolic BP (SBP) and diastolic BP (DBP) can be modelled using both patient characteristics and CMR-derived parameters, which are already collected as part of the standard assessment.

## Methods

### Study cohort

This study included patients from several databases referred to our centre for further assessment of breathlessness. The derivation cohort included 57 patients from the PREFER-CMR registry (ClinicalTrials.gov: NCT05114785) in Norwich, UK and 163 from research registries in Sheffield, UK.^13^ These registries were developed to broadly characterise and subphenotype healthy populations and patients with heart disease by CMR. The inclusion criteria were individuals over the age of 18 undergoing CMR evaluation who provided written informed consent and had a brachial BP measured using a sphygmomanometer before the scan.

**Figure 1:**
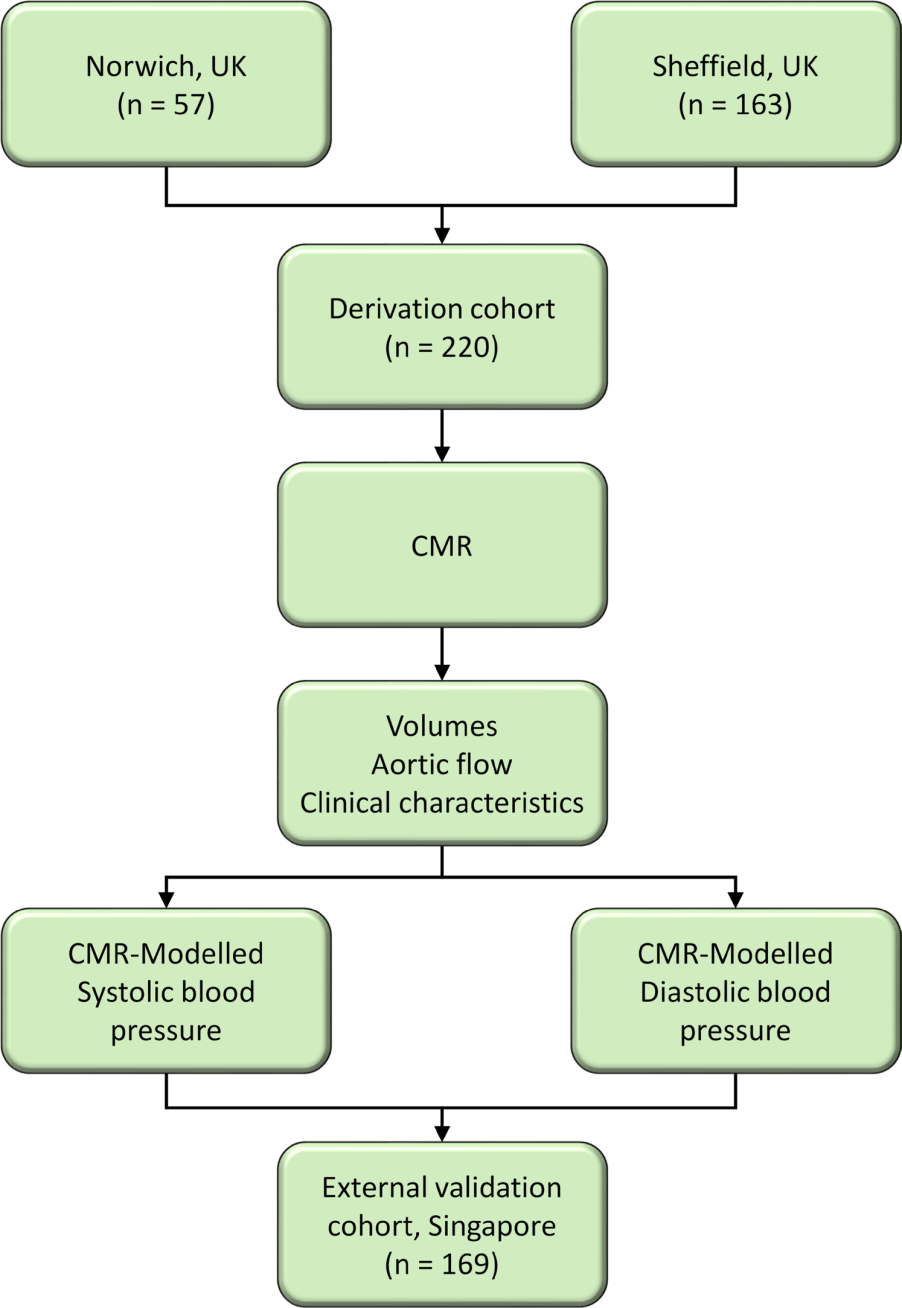
Flow chart illustrating the process of deriving predictive models for systolic and diastolic blood pressures using CMR data. CMR, cardiovascular MR.

To externally validate our findings, we used a cohort of 169 healthy individuals from the INITIATE registry in Singapore (ClinicalTrials.gov: NCT03217240) that recruited both patients and healthy subjects without known cardiovascular disease or cardiovascular risk factors such as hypertension, diabetes and hyperlipidaemia. A flow chart illustrating the recruitment process and the steps taken for deriving predictive models for SBP and DBP is shown in figure 1.

### Cardiac MR protocol

CMR study was performed on a 3.0 Tesla Ingenia (Philips Healthcare, Best, the Netherlands) and 1.5 Tesla Magnetom Sola Siemens system with a superconducting magnet (Siemens Healthineers AG, Erlangen, Germany). All patients were examined in the supine position, head first, using a respiratory sensor and ECG gating. Additionally, the scanner was equipped with a biometric body with 18 coils.

The CMR protocol included baseline survey images and cines and gadolinium enhancement imaging acquisition methods previously described by our group.^14–20^ For standard cines, we acquired 30 phases throughout the cardiac cycle. Other cine acquisition parameters include TR: 2.71, TE: 1.13, field of view (FOV): 360 × 289.3 mm^2^ with phase FOV—80.4%, number of signal averages: 1, matrix: 224×180 (phase), bandwidth: 167.4 kHz, (930 Hz/Px), flip angle: 80, slice thickness: 8 mm and Grappa acceleration with a factor of 2. Additionally, two-dimensional phase contrast CMR data were acquired in the ascending aorta at an orthogonal plane just above the sinotubular junction.

### Cardiac MR analysis

All image analyses were postprocessed with the in-house developed MASS research software (MASS; Version 2022-EXP, Leiden University Medical Center, Leiden, The Netherlands). For left ventricle (LV) and right ventricle (RV) volume analysis, we used the integrated AI-based segmentation tool for endocardial and epicardial borders using the short-axis cine stack of images.^21^ The trabeculae and papillary muscles were included in the analysis. The end-diastolic volum and end-systolic volume were defined as the maximum and minimum values on the volume curves, respectively. LV mass was recorded at the end-diastolic phase.

**Figure 2:**
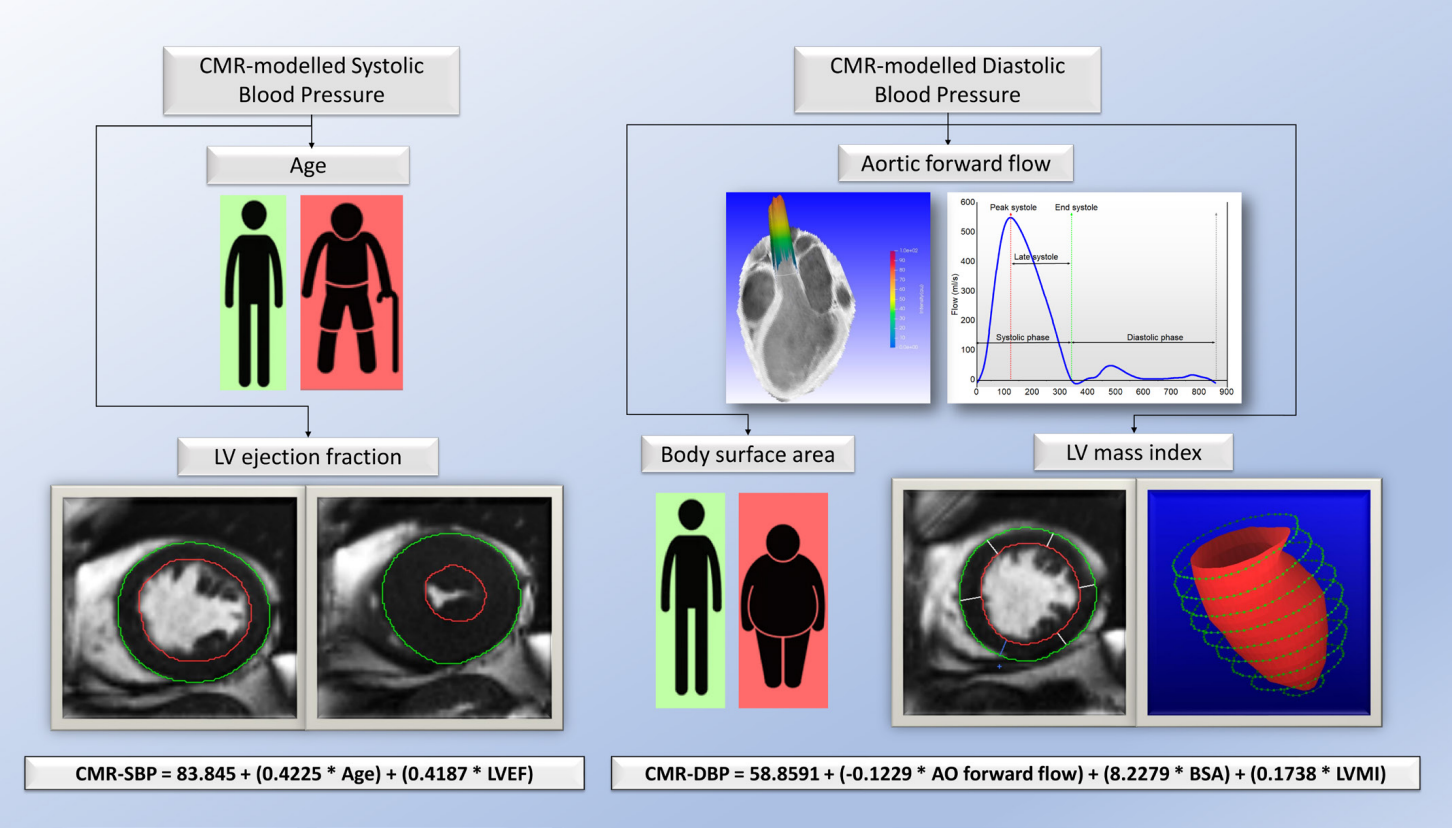
Central illustration demonstrating the demographics and multiparametric CMR volumetric and flow parameters associated with brachial systolic and diastolic blood pressure. CMR, cardiovascular MR.

For flow analysis, we used semiautomated segmentation methods of the ascending and descending aorta throughout the cardiac cycle previously described by our group.^20^ Aortic forward and backward flows were acquired from the resultant flow curve (figure 2). The peak systolic phase was defined as the peak flow rate on the flow curve (figure 2). The calculation of flow displacement involved measuring the distance between the centre point of the vessel and the centre of the velocity of the forward flow. This distance was then adjusted to account for the overall size of the artery during each phase of the cardiac cycle.^22 23^ To calculate the rotational angle (RA), the angle change between the end-systolic point and the point where the flow angle became stable after reaching its peak during systole was measured on the RA curve. Rotational speed (RS) was calculated by dividing the total change in angle by the time interval between consecutive phases. Specifically, the average value of RS was determined from the time after peak systole until the end of systole. The maximal and minimal areas of the ascending aorta were defined by measuring its cross-sectional area throughout the cardiac cycle. The relative area change (RAC) was then calculated as the percentage change between the maximal and minimal areas, using the formula: (maximal area−minimal area)/minimal area×100%.

### Statistical analysis

Data analyses were performed using MedCalc (MedCalc Software, Ostend, Belgium, V.20.215). Normality was assessed using a visual assessment of histograms and the Shapiro-Wilk test. Continuous parametric variables were expressed as mean±SD. Variables that displayed significant correlations (p<0.05) were selected as independent variables for multivariate linear regression analysis, from which a regression model was developed. For comparing CMR-modelled with brachial-measured SBP and DBP, we used Pearson’s correlation coefficient to assess the correlation and Bland-Altman plots to assess agreement and bias. The discriminative capability was evaluated by calculating the area under the curve (AUC) using receiver operating characteristic curves. The significance threshold was set at p<0.05.

## Results

The overall results of the study are summarised in figure 2.

### Study population

Table 1 shows the baseline characteristics of each cohort. Some notable baseline demographic differences were observed, with the validation cohort being statistically younger with a lower body surface area (BSA), a higher proportion of females and a lower mean SBP. The mean DBP was similar between cohorts. Left ventricular CMR parameters were not significantly different, aside from a higher LV Mass Index (LVMI) in the derivation cohort and a slightly higher LV stroke volume index in the validation cohort. Aortic flow parameters were more divergent between the cohorts, with only the AO peak systolic flow and systolic retrograde flow being non-significant between the groups. The validation cohort generally had a greater forward and smaller backward aortic flow with less flow rotation. The derivation cohort had a lower RAC, likely a combination of reduced stroke volume and higher vascular stiffness. The observed differences reflect the focus of the original studies, with the derivation cohort predominantly focused on heart failure with preserved ejection fraction and the validation cohort being healthy, overall resulting in heterogeneity for subsequent modelling.

**Table 1:** Study demographics, CMR functional and aortic flow parameters of the derivation and validation cohorts and their significance

	Derivation cohort (n=220)	Validation cohort (n=169)	P value
Demographics			
Age, years	65±20.5	42±23	<0.0001
Body surface area, m^2^	1.9±0.34	1.71±0.3	<0.0001
Gender, M/F	97/123	96/73	0.01
Systolic blood pressure, mm Hg	133.5±33.5	126±20	<0.0001
Diastolic blood pressure, mm Hg	74.5±15.5	76±16	0.85
CMR functional parameters			
LA volume index, ml/m^2^	37±20	38.4±12	0.34
LV end-diastolic volume index, mL/m^2^	63.8±33.8	71±17	0.55
LV end-systolic volume index, mL/m^2^	21.5±19.2	25.9±10	0.61
LV mass index, g/m^2^	52±20	48±13	<0.0001
LV stroke volume index, mL/m^2^	40±19	44±9	0.04
LV ejection fraction, %	65±17	62±11	0.95
Aortic flow parameters			
AO forward flow, mL	65±38	72.3±20	0.0003
AO backward flow, mL	2±2.6	0.65±1.3	<0.0001
AO peak systolic flow, mL	318±152	349±105	0.12
Systolic retrograde flow, mL	70±41	72±19	0.41
Systolic forward flow, mL	5.6±6.1	3.1±4.2	<0.0001
Average systolic flow displacement, %	24±12	16±8	<0.0001
Rotational angle, °	6.6±14	0±3	0.03
Systolic flow reversal ratio, %	9.2±9.5	4.3±5.5	<0.0001
AO max area, mm^2^	8.4±2.8	6.7±2.3	<0.0001
AO min area, mm^2^	7.2±2.5	5.3±2	<0.0001
Relative area change, %	13.8±8	26.39±20	<0.0001

AO, aorta; CMR, cardiovascular MR; LA, left atrium; LV, Left ventricle.

### Derivation cohort

**Figure 3:**
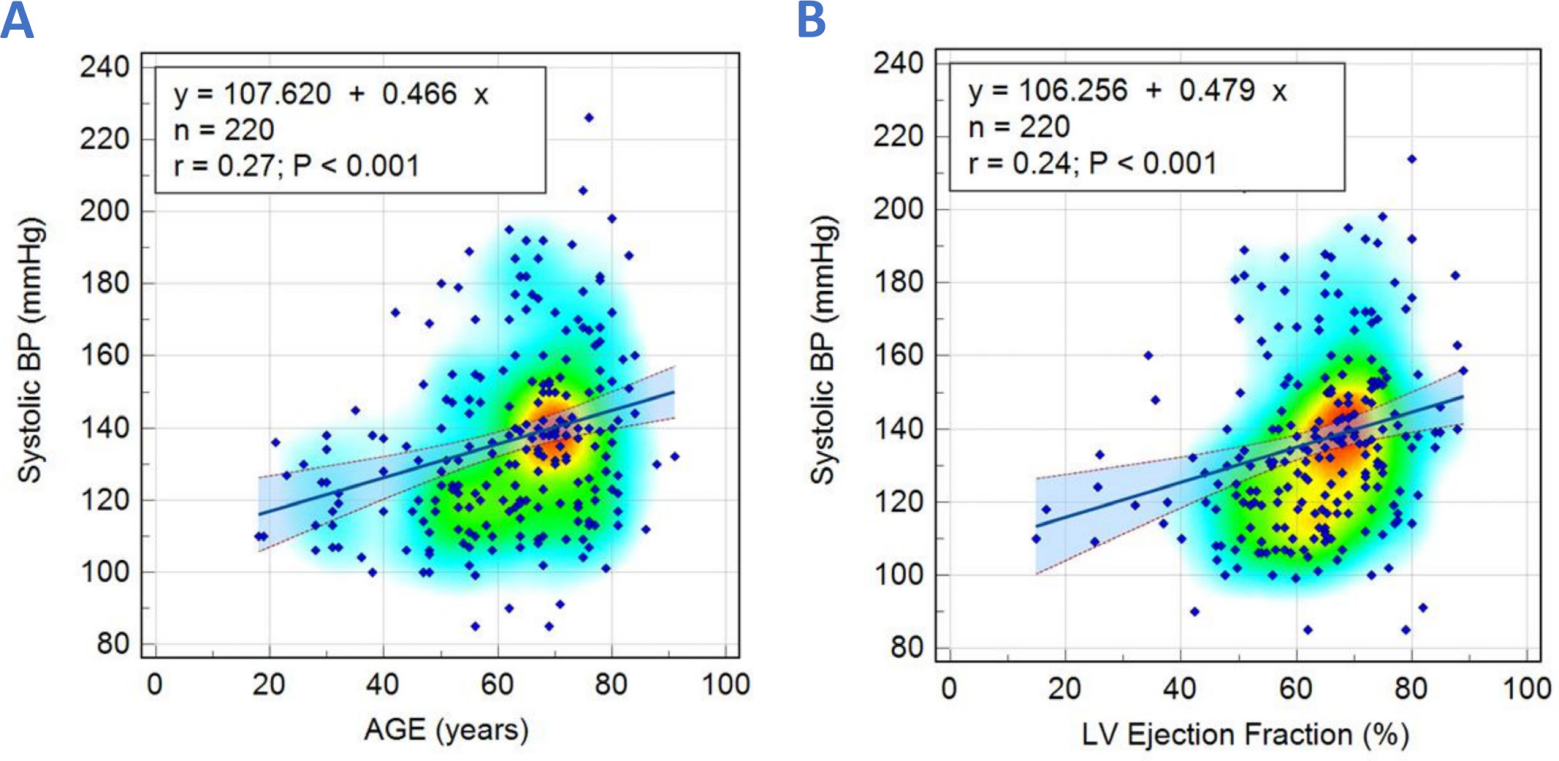
Scatter diagrams with heat maps demonstrating correlations between systolic blood pressure with age (A) and left ventricular (LV) ejection fraction (B).

**Figure 4:**
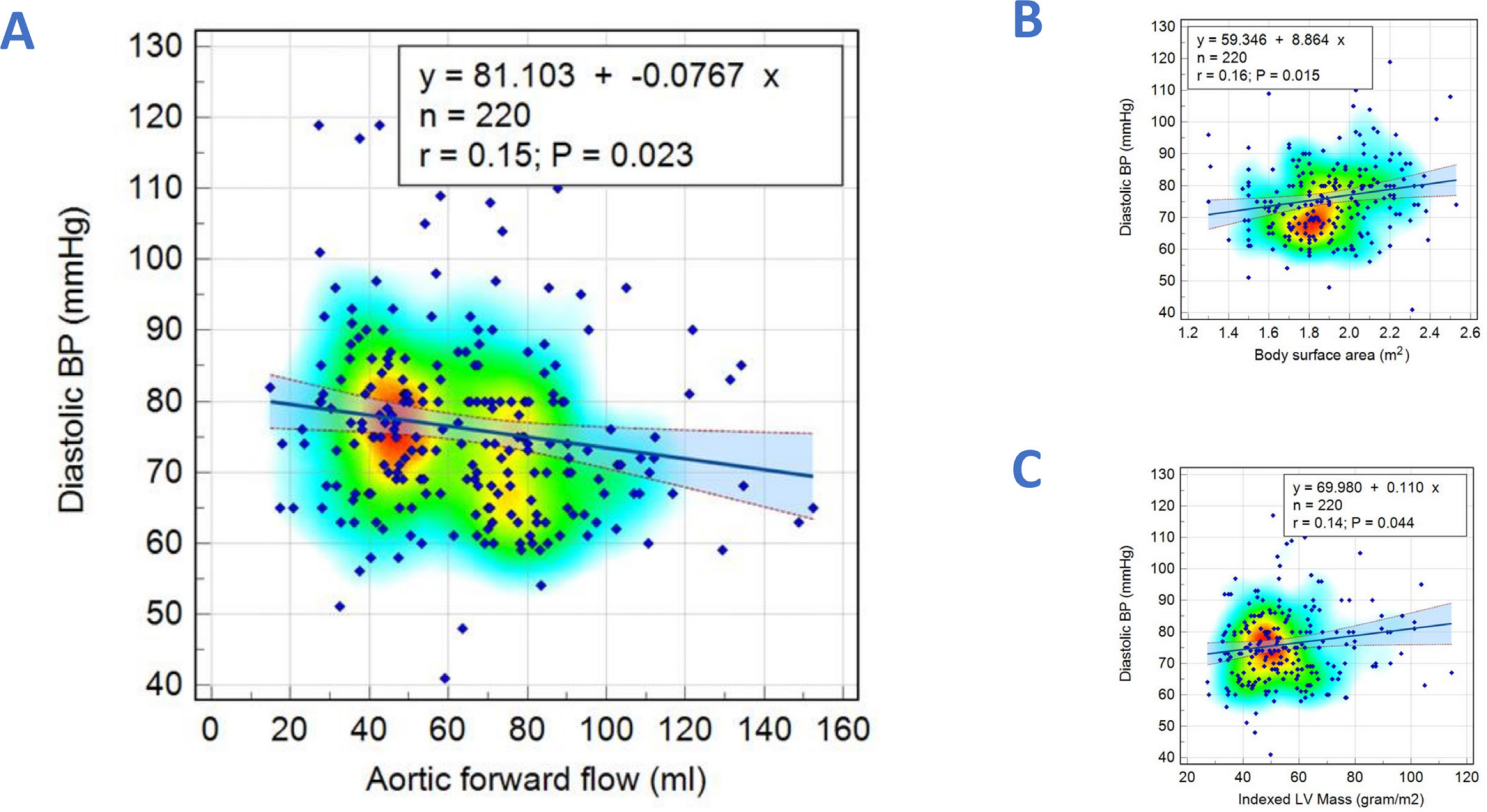
Scatter diagrams with heat maps demonstrating correlations between diastolic blood pressure with aortic forward flow (A), body surface area (B) and Left Ventricular Mass Index (C).

Parameters which were significantly associated with SBP and DBP are shown in table 2. CMR functional and aortic flow parameters that displayed significant correlations with SBP and DBP were selected as independent variables for multivariate linear regression analysis. For SBP, only age (r=0.27, p<0.001) and LV ejection fraction (LVEF) (r=0.24, p<0.001) were found to be significantly correlated (figure 3) (online supplemental table S1). For DBP, only BSA (r=0.16, p=0.01), LVMI (r=0.14, p=0.04) and AO forward flow (r=−0.15, p=0.02) were found to be significantly correlated (figure 4) (online supplemental table S2).

10.1136/openhrt-2023-002484.supp1 Supplementary data

**Table 2:** Correlation coefficient of the study demographics, CMR functional and aortic flow variables with brachial measured systolic and diastolic blood pressures in the derivation cohort

Variable	Systolic blood pressure		Diastolic blood pressure	
	r	P value	r	P value
Age, years	0.27	**0.0001**	0.02	0.81
Body surface area, m^2^	0.05	0.48	0.16	**0.01**
LA volume index, ml/m^2^	0.15	**0.02**	−0.06	0.42
LV end-diastolic volume index, mL/m^2^	−0.13	0.05	−0.05	0.48
LV end-systolic volume index, mL/m^2^	−0.19	**0.005**	0.03	0.70
LV mass index, g/m^2^	0.01	0.89	0.14	**0.04**
LV stroke volume index, ml/m^2^	0.01	0.84	−0.14	**0.03**
LV ejection fraction, %	0.24	**0.0004**	−0.14	**0.04**
AO backward flow, mL	0.06	0.41	0.13	0.06
AO forward flow, mL	−0.07	0.30	−0.15	**0.02**
AO peak systolic flow, mL	−0.11	0.12	−0.08	0.25
Systolic retrograde flow, mL	0.05	0.46	−0.07	0.30
Systolic forward flow, mL	−0.04	0.59	−0.16	**0.01**
Average systolic flow displacement, %	0.20	**0.003**	−0.13	0.06
Rotational angle, °	−0.04	0.59	0.02	0.80
Systolic flow reversal ratio, %	0.07	0.32	−0.01	0.90
AO maximal area, mm^2^	−0.01	0.95	0.07	0.28
AO minimal area, mm^2^	−0.01	0.86	0.10	0.15
Relative area change, %	−0.03	0.70	−0.12	0.07

Bold values denote statistical significance.

AO, aorta; CMR, cardiovascular MR; LA, left atrium; LV, Left ventricle.

A multivariate linear regression model was constructed to predict SBP and DBP. The derived equations were as follows:

CMR-modelled SBP=83.845+(0.4225×age)+(0.4187×LVEF).

CMR-modelled DBP=58.8591+(−0.1229×AO forward flow)+(8.2279×BSA)+(0.1738×LVMI).

### Validation cohort

**Figure 5:**
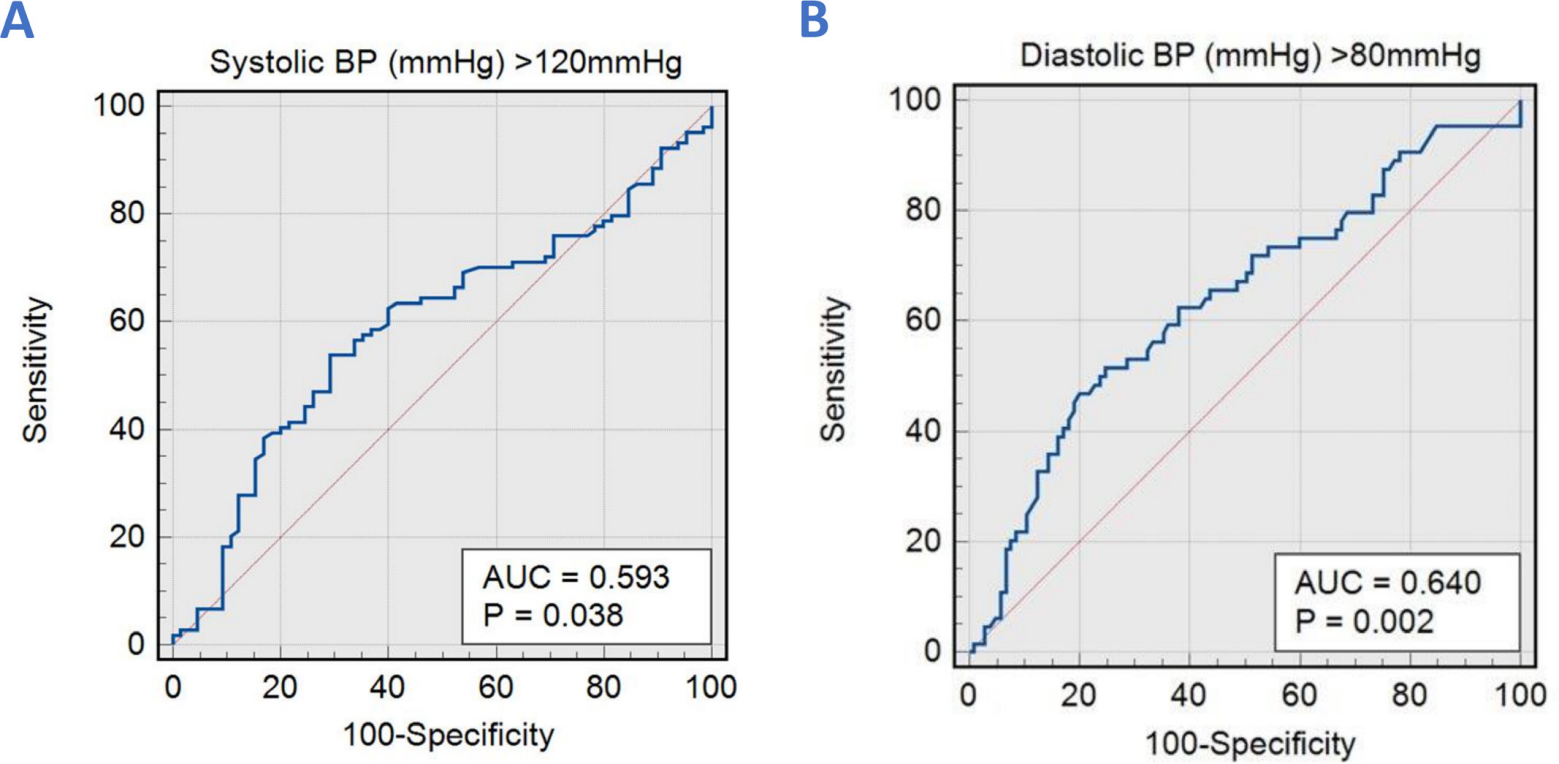
(A) Receiver operator curves demonstrate satisfactory agreement between CMR-modelled and brachial-measured systolic blood pressures (BP) in estimating pressures >120 mmHg. (B) Receiver operator curves show satisfactory agreement between CMR-modelled and brachial-measured diastolic BPs in estimating pressures >80 mmHg. AUC, area under the curve; CMR, cardiovascular MR.

**Figure 6:**
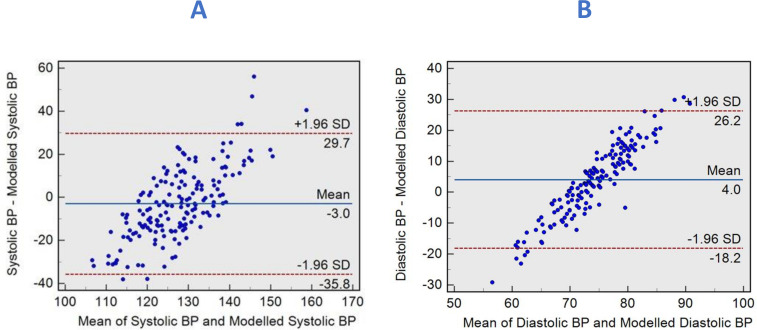
Bland-Altman plots demonstrating the degree of agreement between brachial measured and CMR-modelled systolic blood pressures (BPs) (A) and between brachial measured and CMR-modelled diastolic BP (B). CMR, cardiovascular MR.

We applied the above equations to the validation cohort (n=169) to predict brachial SBP and DBP. The correlation coefficient of the CMR-derived SBP composite model and brachial SBP was (r=0.16, 95% CI 0.011 to 0.305, p=0.03). The diagnostic accuracy (figure 5A) of CMR-derived SBP to predict SBP>120 mmHg had a sensitivity of 54% and a specificity of 71% with an AUC of 0.59 (95% CI 0.74 to 0.89, p=0.038). In Bland-Altman’s analysis, the mean difference between CMR-derived SBP and brachial-measured SBP was −3 mmHg, with limits of agreement (LOA) of −35.8 to 29.7 mmHg (figure 6A).

Similarly, with DBP, the correlation coefficient of the CMR-derived DBP composite model and brachial DBP were (r=0.27, 95% CI 0.122 to 0.403, p=0.0004). The diagnostic accuracy (figure 5B) of CMR-derived DBP to predict brachial DBP>80 mmHg had a sensitivity of 47% and a specificity of 81% with an AUC of 0.64 (95% CI 0.56 to 0.71, p=0.002). Moreover, the mean bias between CMR-derived DBP and brachial-measured DBP was 4 mmHg, with LOA of −18.2 to 26.2 mmHg (figure 6B).

## Discussion

This study aimed to develop and test a physiological model to estimate SBP and DBPs using patient characteristics and CMR functional and flow data. Our findings suggest that a model incorporating patient demographics, LVEF, left ventricular mass index and aortic forward flow can accurately predict SBP and DBP. CMR-modelled SBP and DBP exhibit modest correlation with brachial measurements, especially with systolic pressures of >120 mmHg and diastolic pressures of >80 mmHg. Furthermore, our model performed well in an external validation cohort from a different centre, indicating its potential robustness in heterogeneous patient populations.

Developing a reliable and accurate model to estimate BP using non-invasive variables, including patient demographics and CMR imaging data, has significant clinical advantages. It could provide haemodynamic indices for CMR imaging, which would aid the assessment of afterload-dependent pathologies such as valvular heart disease and heart failure. We have previously reported on non-invasive CMR-derived pulmonary capillary wedge pressure, itself an index of LV end-diastolic pressure, and therefore, providing an index of preload.^13^ Therefore, CMR can provide both preload and afterload assessment to interpret contractility.

The present model could be retrospectively applied where brachial BP measurements were not obtained prior to the study. Additionally, the ability to estimate BP without additional measurements would save time, reduce patient discomfort and minimise errors from transient BP fluctuation.

Elevated BP is a leading risk factor for cardiovascular diseases and a significant cause of morbidity and mortality worldwide. The ability to estimate pressure using imaging data has been investigated in several studies using different imaging modalities, including MRI,^24–26^ ultrasound^27^ and CT.^28–30^ CMR imaging is particularly well suited for estimating BP due to its high spatial and temporal resolution and the ability to measure both flow and function.

Our study used a multiparametric approach to estimate BP, including demographic variables (age and body mass index) and CMR-derived functional and flow parameters, including LVEF, LVMI and AO forward flow. Several studies have investigated the relationship between CMR-derived parameters and BP in different patient populations. For example, studies have shown that LVMI, left atrial volume index, and aortic distensibility are strongly associated with BP in patients with hypertension.^31–33^ Our study similarly shows that LVMI correlated with SBP. BP has previously been shown to affect aortic and mitral regurgitation severity, which are dynamic valvular incompetencies.^34 35^ These studies highlight the importance of accurate BP estimation in assessing cardiovascular diseases.

### Limitations

Although we included patients from multiple databases, a limitation of our study is the relatively small sample size of the derivation cohort, which could limit the generalisability of our results. Furthermore, patients included in the study were either healthy volunteers or those with cardiovascular diseases. The variations in demographics and clinical parameters, such as age, gender distribution and SBP, between the two cohorts might introduce some level of heterogeneity that could potentially impact the generalisability of our findings. It is unclear whether our model would perform similarly in other patient populations, such as those with renal or pulmonary diseases. Therefore, the transferability of our results to broader populations should be interpreted with caution, as our findings may have specific relevance to cohorts with similar characteristics. Future studies should aim to address this limitation by using more homogeneous populations, where possible, to validate further and extend the applicability of our results. Brachial BP measurements were taken before the CMR scan in the resting state so that they reflected the haemodynamics during imaging assessment and would be akin to a clinic BP recording. They do not necessarily reflect the average long-term or ambulatory BP. They might also be subject to fluctuations such as those due to white-coat syndrome or anxiety regarding CMR assessment on the day.

## Conclusion

In conclusion, our study demonstrates that a CMR-modelled SBP and DBP can estimate BP using patient demographics and CMR-derived functional and flow data. This model has the potential to provide afterload assessment during CMR imaging and could aid in the management of complex cardiovascular diseases. Furthermore, the ability to estimate BP without additional measurements would save time, could be retrospectively applied, reduce patient discomfort and minimise errors. Future studies are needed to validate our findings in larger patient populations and also with ambulatory BP assessments.

## Data Availability

Data are available on reasonable request. The datasets generated and analysed during the current study are not publicly available. Access to the raw images of patients is not permitted since specialised postprocessing imaging-based solutions can identify the study patients in the future. Data are available from the corresponding author on reasonable request.

## References

[1] Mills KT, Stefanescu A, He J. The global epidemiology of hypertension. Nat Rev Nephrol 2020;16:223–37. 10.1038/s41581-019-0244-2 32024986 PMC7998524

[2] Li Y, Chan E, Puyol-Antón E, et al. Hemodynamic determinants of elevated blood pressure and hypertension in the middle to older-age UK population: a UK Biobank imaging study. Hypertension 2023;80:2473–84. 10.1161/HYPERTENSIONAHA.122.20969 37675583 PMC10876164

[3] Ricci F, Fedorowski A, Radico F, et al. Cardiovascular morbidity and mortality related to orthostatic hypotension: a meta-analysis of prospective observational studies. Eur Heart J 2015;36:1609–17. 10.1093/eurheartj/ehv093 25852216

[4] Bahat G, Ilhan B, Tufan A, et al. Hypotension in nursing home residents on antihypertensive treatment: is it associated with mortality? J Am Med Dir Assoc 2021;22:2319–24. 10.1016/j.jamda.2021.03.004 33848503

[5] Hayek A, Derimay F, Green L, et al. Impact of arterial blood pressure on ultrasound hemodynamic assessment of aortic valve stenosis severity. J Am Soc Echocardiogr 2020;33:1324–33. 10.1016/j.echo.2020.06.013 32868157

[6] Akintunde AA, Akinwusi PO, Familoni OB, et al. Effect of systemic hypertension on right ventricular morphology and function: an echocardiographic study. Cardiovasc J Afr 2010;21:252–6. 10.5830/cvja-2010-013 20972511 PMC3721898

[7] Pérez Del Villar C, Savvatis K, López B, et al. Impact of acute hypertension transients on diastolic function in patients with heart failure with preserved ejection fraction. Cardiovasc Res 2017;113:906–14. 10.1093/cvr/cvx047 28402411

[8] Tsimploulis A, Lam PH, Arundel C, et al. Systolic blood pressure and outcomes in patients with heart failure with preserved ejection fraction. JAMA Cardiol 2018;3:288–97. 10.1001/jamacardio.2017.5365 29450487 PMC5875342

[9] Das P, Pocock C, Chambers J. The patient with a systolic murmur: severe aortic stenosis may be missed during cardiovascular examination. QJM 2000;93:685–8. 10.1093/qjmed/93.10.685 11029480

[10] Baumgartner H, Hung J, Bermejo J, et al. Recommendations on the echocardiographic assessment of aortic valve stenosis: a focused update from the European Association of cardiovascular imaging and the American society of echocardiography. Eur Heart J Cardiovasc Imaging 2017;18:254–75. 10.1093/ehjci/jew335 28363204

[11] Zoghbi WA, Adams D, Bonow RO, et al. Recommendations for noninvasive evaluation of native valvular regurgitation: a report from the American society of echocardiography developed in collaboration with the society for cardiovascular magnetic resonance. J Am Soc Echocardiogr 2017;30:303–71. 10.1016/j.echo.2017.01.007 28314623

[12] Muntner P, Einhorn PT, Cushman WC, et al. Blood pressure assessment in adults in clinical practice and clinic-based research: JACC scientific expert panel. J Am Coll Cardiol 2019;73:317–35. 10.1016/j.jacc.2018.10.069 30678763 PMC6573014

[13] Garg P, Gosling R, Swoboda P, et al. Cardiac magnetic resonance identifies raised left ventricular filling pressure: prognostic implications. Eur Heart J 2022;43:2511–22. 10.1093/eurheartj/ehac207 35512290 PMC9259376

[14] Assadi H, Uthayachandran B, Li R, et al. Kat-ARC accelerated 4D flow CMR: clinical validation for transvalvular flow and peak velocity assessment. Eur Radiol Exp 2022;6:46. 10.1186/s41747-022-00299-5 36131185 PMC9492816

[15] Assadi H, Grafton-Clarke C, Demirkiran A, et al. Mitral regurgitation quantified by CMR 4D-flow is associated with microvascular obstruction post reperfused ST-segment elevation myocardial infarction. BMC Res Notes 2022;15:181. 10.1186/s13104-022-06063-7 35570318 PMC9107700

[16] Garg P, Broadbent DA, Swoboda PP, et al. Acute infarct extracellular volume mapping to quantify myocardial area at risk and chronic infarct size on cardiovascular magnetic resonance imaging. Circ Cardiovasc Imaging 2017;10:e006182. 10.1161/CIRCIMAGING.117.006182 28674085

[17] Garg P, van der Geest RJ, Swoboda PP, et al. Left ventricular thrombus formation in myocardial infarction is associated with altered left ventricular blood flow energetics. Eur Heart J Cardiovasc Imaging 2019;20:108–17. 10.1093/ehjci/jey121 30137274 PMC6302263

[18] Garg P, Crandon S, Swoboda PP, et al. Left ventricular blood flow kinetic energy after myocardial infarction - insights from 4D flow cardiovascular magnetic resonance. J Cardiovasc Magn Reson 2018;20:61. 10.1186/s12968-018-0483-6 30165869 PMC6117925

[19] Li R, Assadi H, Matthews G, et al. The importance of mitral valve prolapse doming volume in the assessment of left ventricular stroke volume with cardiac MRI. Med Sci (Basel) 2023;11:13. 10.3390/medsci11010013 36810480 PMC9945133

[20] Zhao X, Garg P, Assadi H, et al. Aortic flow is associated with aging and exercise capacity. Eur Heart J Open 2023;3:oead079. 10.1093/ehjopen/oead079 37635784 PMC10460199

[21] Alabed S, Alandejani F, Dwivedi K, et al. Validation of artificial intelligence cardiac MRI measurements: relationship to heart catheterization and mortality prediction. Radiology 2022;305:68–79. 10.1148/radiol.212929 35699578 PMC9527336

[22] Burris NS, Sigovan M, Knauer HA, et al. Systolic flow displacement correlates with future ascending aortic growth in patients with bicuspid aortic valves undergoing magnetic resonance surveillance. Invest Radiol 2014;49:635–9. 10.1097/RLI.0000000000000064 24784460

[23] Sigovan M, Hope MD, Dyverfeldt P, et al. Comparison of four-dimensional flow parameters for quantification of flow eccentricity in the ascending aorta. J Magn Reson Imaging 2011;34:1226–30. 10.1002/jmri.22800 21928387

[24] Kazemi A, Padgett DA, Callahan S, et al. Relative pressure estimation from 4D flow MRI using generalized Bernoulli equation in a phantom model of arterial stenosis. Magn Reson Mater Phy 2022;35:733–48. 10.1007/s10334-022-01001-x 35175449

[25] Zhang J, Brindise MC, Rothenberger S, et al. 4D flow MRI pressure estimation using velocity measurement-error-based weighted least-squares. IEEE Trans Med Imaging 2020;39:1668–80. 10.1109/TMI.2019.2954697 31751234 PMC10359051

[26] Donati F, Figueroa CA, Smith NP, et al. Non-invasive pressure difference estimation from PC-MRI using the work-energy equation. Med Image Anal 2015;26:159–72. 10.1016/j.media.2015.08.012 26409245 PMC4686008

[27] Zakrzewski AM, Anthony BW. Noninvasive blood pressure estimation using ultrasound and simple finite element models. IEEE Trans Biomed Eng 2018;65:2011–22. 10.1109/TBME.2017.2714666 28613159 PMC10615346

[28] Pappu S, Lerma J, Khraishi T. Brain CT to assess intracranial pressure in patients with traumatic brain injury. J Neuroimaging 2016;26:37–40. 10.1111/jon.12289 26752449

[29] Mizutani T, Manaka S, Tsutsumi H. Estimation of intracranial pressure using computed tomography scan findings in patients with severe head injury. Surg Neurol 1990;33:178–84. 10.1016/0090-3019(90)90181-n 2315829

[30] Li M, Wang S, Lin W, et al. Cardiovascular parameters of chest CT scan in estimating pulmonary arterial pressure in patients with pulmonary hypertension. Clin Respir J 2018;12:572–9. 10.1111/crj.12564 27696745

[31] Milan A, Caserta MA, Dematteis A, et al. Blood pressure levels, left ventricular mass and function are correlated with left atrial volume in mild to moderate hypertensive patients. J Hum Hypertens 2009;23:743–50. 10.1038/jhh.2009.15 19262581

[32] Urbina EM, Mendizábal B, Becker RC, et al. Association of blood pressure level with left ventricular mass in adolescents. Hypertension 2019;74:590–6. 10.1161/HYPERTENSIONAHA.119.13027 31327264

[33] Kaess BM, Rong J, Larson MG, et al. Aortic stiffness, blood pressure progression, and incident hypertension. JAMA 2012;308:875–81. 10.1001/2012.jama.10503 22948697 PMC3594687

[34] Nazarzadeh M, Pinho-Gomes A-C, Smith Byrne K, et al. Systolic blood pressure and risk of valvular heart disease: a mendelian randomization study. JAMA Cardiol 2019;4:788–95. 10.1001/jamacardio.2019.2202 31290937 PMC6624812

[35] Rahimi K, Mohseni H, Otto CM, et al. Elevated blood pressure and risk of mitral regurgitation: a longitudinal cohort study of 5.5 million United Kingdom adults. PLoS Med 2017;14:e1002404. 10.1371/journal.pmed.1002404 29040269 PMC5644976

