# Supplementary material for: Cardiac MR modelling of systolic and diastolic blood pressure
Source: Open Heart. 2023 Dec 18;10(2):e002484. doi: 10.1136/openhrt-2023-002484 (PMC10749049; doi:10.1136/openhrt-2023-002484)
Supplement: Supplementary data [file openhrt-2023-002484supp001.pdf]

Supplementary tables

Table S1 – Stepwise multivariable linear regression analysis for systolic blood pressure.

Dependent Y	**SBP**					
Least squares multiple regression						
Method	Stepwise					
Enter variable if P<	0.05					
Remove variable if P>	0.1					
Sample size	220					
Coefficient of determination R2	0.1169					
R2-adjusted	0.1088					
Multiple correlation coefficient	0.342					
Residual standard deviation	24.291					
Regression Equation						
Independent variables	Coefficient	Std. Error	t	P	rpartial	rsemipartial
**(Constant)**	83.845					
**Age**	0.4225	0.11	3.84	0.0002	0.252	0.2
**LV ejection fraction**	0.4187	0.1291	3.24	0.001	0.215	0.2
Variables not included in the model						
**LA volume index**						
**LV end-diastolic volume index**						
**LV end-systolic volume index**						
**Average systolic flow displacement**						
Analysis of Variance						
Source	DF	Sum of Squares	Mean Square			
Regression	2	16957	8479			
Residual	217	128043	590			
F-ratio	14.369					
Significance level	P<0.0001					

Assadi H, *et al. Open Heart* 2023; 10:e002484. doi: 10.1136/openhrt-2023-002484

BMJ Publishing Group Limited (BMJ) disclaims all liability and responsibility arising from any reliance placed on this supplemental material which has been supplied by the author(s)

Table S2 – Stepwise multivariable linear regression analysis for diastolic blood pressure.

Dependent Y	**DBP**					
Least squares multiple regression						
Method	Stepwise					
Enter variable if P<	0.05					
Remove variable if P>	0.1					
Sample size	220					
Coefficient of determination R2	0.09157					
R2-adjusted	0.07896					
Multiple correlation coefficient	0.3026					
Residual standard deviation	12.7421					
Regression Equation						
Independent variables	Coefficient	Std. Error	t	P	rpartial	rsemipartial
**(Constant)**	58.8591					
**Body surface area**	8.2279	3.538	2.326	0.021	0.156	0.1508
**Aortic forward flow**	-0.1229	0.03532	-3.479	0.0006	-0.230	0.2256
**LV mass index**	0.1738	0.05738	3.028	0.0028	0.201	0.1964
Variables not included in the model						
**LV ejection fraction**						
**LV stroke volume index**						
**Average systolic flow displacement**						
**Systolic forward flow**						
**Aortic backward flow**						
Analysis of Variance						
Source	DF	Sum of Squares	Mean Square			
Regression	3	3535.19	1178.4			
Residual	216	35069.7	162.36			
F-ratio	7.2579					
Significance level	P=0.0001					
